# Evidence for the activation of pyroptotic and apoptotic pathways in RPE cells associated with NLRP3 inflammasome in the rodent eye

**DOI:** 10.1186/s12974-018-1062-3

**Published:** 2018-01-12

**Authors:** Jiangyuan Gao, Jing Z. Cui, Eleanor To, Sijia Cao, Joanne A. Matsubara

**Affiliations:** Department of Ophthalmology and Visual Sciences, Eye Care Centre, Faculty of Medicine, University of British Columbia, 2550 Willow Street, Vancouver, BC V5Z 3N9 Canada

**Keywords:** Age-related macular degeneration, Retinal pigment epithelium, NLRP3 inflammasome, Cell death, Amyloid beta

## Abstract

**Background:**

Age-related macular degeneration (AMD) is a devastating eye disease causing irreversible vision loss in the elderly. Retinal pigment epithelium (RPE), the primary cell type that is afflicted in AMD, undergoes programmed cell death in the late stages of the disease. However, the exact mechanisms for RPE degeneration in AMD are still unresolved. The prevailing theories consider that each cell death pathway works independently and without regulation of each other. Building upon our previous work in which we induced a short burst of inflammasome activity in vivo, we now investigate the effects of prolonged inflammasome activity on RPE cell death mechanisms in rats.

**Methods:**

Long-Evans rats received three intravitreal injections of amyloid beta (Aβ), once every 4 days, and were sacrificed at day 14. The vitreous samples were collected to assess the levels of secreted cytokines. The inflammasome activity was evaluated by both immunohistochemistry and western blot. The types of RPE cell death mechanisms were determined using specific cell death markers and morphological characterizations.

**Results:**

We found robust inflammasome activation evident by enhanced caspase-1 immunoreactivity, augmented NF-κB nuclear translocalization, increased IL-1β vitreal secretion, and IL-18 protein levels. Moreover, we observed elevated proteolytic cleavage of caspase-3 and gasdermin D, markers for apoptosis and pyroptosis, respectively, in RPE-choroid tissues. There was also a significant reduction in the anti-apoptotic factor, X-linked inhibitor of apoptosis protein, consistent with the overall changes of RPE cells. Morphological analysis showed phenotypic characteristics of pyroptosis including RPE cell swelling.

**Conclusions:**

Our data suggest that two cell death pathways, pyroptosis and apoptosis, were activated in RPE cells after exposure to prolonged inflammasome activation, induced by a drusen component, Aβ. The involvement of two distinct cell death pathways in RPE sheds light on the potential interplay between these pathways and provides insights on the future development of therapeutic strategies for AMD.

**Electronic supplementary material:**

The online version of this article (10.1186/s12974-018-1062-3) contains supplementary material, which is available to authorized users.

## Background

Age-related macular degeneration (AMD) is a neurodegenerative disease that strikes the macula, causing irreversible blindness to people over the age of 50 in industrialized countries. The global prevalence of AMD has created substantial economic and social burden with a projected estimate of 196 million people living with AMD in 2020 [1]. Clearly, better eye care strategies need to be designed to provide health care services to those in need. Clinically, AMD can be predicted by a severity scale that is a function of drusen deposition and pigment abnormalities [2]. The accumulation of drusen both in size and in number has become a hallmark of AMD progression. As it progresses, AMD transitions from early benign stages into advanced vision-threatening stages, presenting with either choroidal neovascularization (CNV, wet form) and/or geographic atrophy (GA, dry form). Although CNV is the severe subtype of the advanced AMD, it is clinically managed using the anti-vascular endothelial growth factor (VEGF) therapy [3]. However, there is no effective treatment to slow down the more prevalent dry form, which makes up approximately 90% of all AMD cases. Retinal pigment epithelium (RPE) cell death and secondary photoreceptor degeneration are two signature changes that lead to central vision loss in GA, the advanced stage of dry AMD. Hence, it is paramount to understand the fundamental mechanisms underlying these devastating impacts to the retina, especially the ones that undermine RPE health.

Despite the lack of consensus on the exact cell death pathway(s) involved, there have been three candidate cell death mechanisms proposed to underlie RPE atrophy in GA, including necrosis, apoptosis, and pyroptosis [4–6]. Necrosis is a classic form of RPE cell death in GA, which was reported in earlier clinical-pathological studies by Sarks et al. [4], and also in basic research projects with ultrastructural and histochemical data [7]. Apoptotic RPE cell death, on the other hand, has gained substantial support from the literature in recent years. Using postmortem human eyes, Kaneko and colleagues identified the activation of caspase-3 in the RPE layer of GA eyes, but not in normal control eyes [5]. Moreover, Dunaief et al. demonstrated statistically significant differences in the number of terminal deoxynucleotidyl transferase dUTP nick end-labeling (TUNEL) positive retinal cells in postmortem retinas with AMD, compared to normal controls [8]. The third proposed mechanism of RPE death is pyroptosis, which is an inflammatory form of programmed cell death [9]. The cornerstone of pyroptosis is the activation of an intracellular multi-protein complex named the inflammasome. NLR family pyrin domain containing 3 (NLRP3) inflammasome is the most widely studied machinery and consists of NLRP3, active caspase-1, and a bridging adaptor, apoptosis-associated speck-like protein containing a carboxy-terminal CARD (ASC). For the NLRP3 inflammasome to function, it requires sequential treatment of two types of pathological stimuli, (1) a priming signal to activate nuclear factor kappa B (NF-κB) and upregulate the transcription of NLRP3 and interleukin (IL)-1β precursor protein; (2) an activation signal to trigger the inflammasome assembly for the production of two pro-inflammatory cytokines, IL-1β and IL-18. The relationship between NLRP3 inflammasome and AMD pathology has been an attractive subject in the field, and current knowledge on this subject is reviewed elsewhere in detail [10]. Although all three cell death pathways described above seems to govern RPE cell fate in GA to some extent, it is still unclear whether these mechanisms act independently or in synergy.

Earlier work, including ours, demonstrated that components found in drusen, (e.g.*,* amyloid beta, complement cascade products) increase in the aged retina [11, 12]. Previously, we have established a rat intraocular injection model to mimic the increasing amyloid beta (Aβ) load associated with drusen in human eyes [13]. We demonstrated that drusen component, Aβ, triggers a short lasting pro-inflammatory response in RPE via the activation of NF-κB and NLRP3 inflammasome [13, 14], which can be specifically abolished by vinpocetine (an NF-κB inhibitor) [15]. Intriguingly, RPE cell loss is not a feature of the short lasting pro-inflammation associated with the acute Aβ intravitreal model [13]. In the present study, we extended the duration of outer retina pro-inflammation by making sequential Aβ injections, in order to better model the chronic pro-inflammatory events and associated cell death underlying the pathogenesis of the dry form of AMD.

## Methods

### Preparation of oligomeric Aβ

The lyophilized, synthetic Aβ1–40 peptide (hereafter referred to as “Aβ”) in the HCl salt form was purchased from American Peptide (Sunnyvale, CA). We chose Aβ1–40 peptide over its structurally similar but more toxic, Alzheimer’s disease (AD)-specific, form of Aβ1–42 peptide based on earlier studies that demonstrated the presence of Aβ1–40 in drusen deposits in postmortem human eyes [13]. Oligomeric Aβ was prepared according to published protocols [16, 17]. Briefly, the synthetic Aβ peptide was first reconstituted in 1,1,1,3,3,3-hexafluoro-2-propanol (HFIP, Sigma Aldrich, St. Louis, MO) and was evaporated by speed vacuum, resulting in thin transparent Aβ peptide film. The Aβ peptide film was then reconstituted in 100% dimethyl sulfoxide (DMSO, Sigma Aldrich) to a concentration of 1 mM, and further diluted in pre-warmed phosphate buffered saline (PBS, pH 7.4) to produce the Aβ injection solution of 323 μM, equivalent to 1.4 μg/μL. The injection solution was subsequently incubated at 37 °C for 48 h to form the oligomeric Aβ. Confirmation of successful oligomeric Aβ formation was achieved by western blot (WB) using our published protocols [11]. Reverse Aβ40–1 peptide (hereafter, referred to as “control”) served as a sequence control for Aβ and was prepared in the same fashion. The absence of protein bands recognized by a mouse monoclonal anti-Aβ1–16 antibody (clone 6E10, Table 1) in the control solution indicated proper preparation (Additional file 1: Figure S1).

**Table 1:** List of primary antibodies

Antigen	Antibody	Dilution	Source	Applications
Amyloid-beta amino acid 1-16 (Aβ_1–16_)	Mouse monoclonal anti-Aβ_1–16_ (clone 6E10)	1:2000 1:500	BioLegend, Dedham, MA	Western blot Immunohistochemistry
Caspase-1	Rabbit monoclonal	1:300	Abcam, Cambridge, UK	Immunohistochemistry
Phosphorylated NF-κB p65 (Ser 276)	Rabbit polyclonal	1:75	Santa Cruz Biotechnology, Dallas, TX	Immunohistochemistry
Interleukin-18 (IL-18)	Rabbit polyclonal	1:100 1:1000	Santa Cruz Biotechnology, Dallas, TX	Immunohistochemistry Western blot
Active caspase-3 (aCasp-3)	Rabbit monoclonal	1:1000	Cell Signaling Technology, Beverly, MA	Immunohistochemistry
X-linked inhibitor of apoptosis (XIAP)	Mouse monoclonal	1:1000	BD Transduction Laboratories, San Jose, CA	Western blot
Gasdermin D (GSDMD)	Mouse monoclonal	1:100	Santa Cruz Biotechnology, Dallas, TX	Western blot
GAPDH	Mouse monoclonal	1:10,000	EMD Millipore, Billerica, MA	Western blot

### Animals

Adult female Long-Evans rats at the age of 4.5 month (Charles River, Wilmington, MA) were randomly divided into two groups. Group 1 (*N* = 16) comprised rats receiving intravitreal injections of Aβ (5 μL at 1.4 μg/μL as previously published [13]) once every 4 days for a total of three injections. Group 2 (*N* = 16) rats received intravitreal injections of the control solution (reverse Aβ40–1 peptide) the same way as described for group 1. All rats were sacrificed on the 14th day after initial injection (day 14). Eyes were immediately enucleated and frozen for WB, polymerase chain reaction (PCR), and enzyme-linked immunosorbent assay (ELISA) or fixed in 4% paraformaldehyde diluted in Dulbecco’s PBS (Invitrogen, Carlsbad, CA) for 48–72 h prior to paraffin embedding.

### Immunohistochemistry (caspase-1, IL-18, NF-κB, active caspase-3)

Paraffin-embedded rat eye tissues were processed following established protocols [13]. Sections from both the Aβ and the control groups were processed simultaneously in an effort to minimize variability in immunoreactivity conditions (*N* = 3 per group). Primary antibodies recognizing total caspase-1, IL-18, NF-κB, and active caspase-3 are described in Table 1. Non-specific isotype IgGs (Sigma Aldrich) matching the species of primary antibodies are used on negative control tissue sections. For visualization, the slides were developed using the Vector® AEC substrate kit (Vector Laboratories, Burlington ON, Canada) and were counterstained with Mayer’s Hematoxylin (Sigma Aldrich) for the nuclei. Sections processed simultaneously were analyzed and scored so as to avoid difference in immunostaining due to conditions such as temperature and stock of antibodies. Caspase-1, IL-18, and active caspase-3 immunoreactivity was scored in a masked fashion and semi-quantitatively based on a 0–3 point scale. A score of 0 indicates no detectable staining above the background level as compared to the negative control sections, whereas a score of 1, 2, or 3 suggests weak, intermediate, and robust intensity of the immunoreactivity, respectively. The immunoreactivity scores of caspase-1, IL-18, and active caspase-3 were averaged and normalized to the control group.

To detect NF-κB translocalization, an antibody recognizing the phosphorylated Ser 276 locus on NF-κB p65 subunit was used (Table 1). Immunoreactivity was measured quantitatively, in a masked fashion, using a × 60 objective lens and × 10 eyepieces. Positive RPE nuclei were identified as containing both the red AEC chromogen and blue hematoxylin counterstain, thus resulting in a purple appearance distinct from the unlabeled RPE nuclei that were blue in color due to the hematoxylin counterstain alone. The number of NF-κB positive nuclei was converted to percentage of all RPE nuclei in the sample area and was normalized to the control group.

### Suspension array for vitreal cytokines

An ELISA-based cytokine assay for vitreal cytokines was carried out (Bio-Plex 200 System, Bio-Rad Laboratories, Hercules, CA). The assay targeted the following cytokines: erythropoietin (EPO), granulocyte colony stimulating factor (G-CSF), granulocyte macrophage colony stimulating factor (GM-CSF), chemokine (C-X-C motif) ligand 1 (GRO/KC), interferon-gamma (IFN-γ), IL-1α, IL-1β, IL-2, IL-4, IL-5, IL-6, IL-7, IL-10, IL-12p70, IL-13, IL-17, IL-18, macrophage colony stimulating factor (M-CSF), macrophage inflammatory protein 1alpha (MIP-1α), MIP-3α, regulated on activation, normal T cell expressed and secreted (RANTES), TNF-α, and vascular endothelial growth factor (VEGF). Vitreous from rat eyes in the same group (either Aβ or control) were pooled (*N* = 7). Experiments were carried out following methods in our earlier publication [15].

### Western blot

To determine the level of X-linked inhibitor of apoptosis (XIAP), whole retina tissues (including neuroretina, RPE, Bruch’s membrane, and choroid) from each of the two injection groups were used (*N* = 5). To investigate IL-18 secretion and gasdermin D (GSDMD) cleavage, RPE-choroid tissues from each of the two injection groups were dissected out and pooled to use (*N* ≥ 3). Tissues were homogenized in ice-cold RIPA buffer (Thermo Fisher Scientific, Waltham, MA) containing protease inhibitor cocktail (Roche Diagnostics, Indianapolis, IN). Protein lysates were run under reducing conditions and established blotting procedures were followed. Detailed information on the primary antibodies used in western blotting can be found in Table 1 [11]. As an internal protein loading control, GAPDH was detected either on the stripped membrane or using freshly thawed protein lysates (Table 1). The protein band intensity of XIAP (57 kDa), IL-18 (18 kDa), pro-GSDMD (53 kDa), N-GSDMD (30 kDa), and GAPDH (36 kDa) was individually measured using ImageJ (NIH, Bethesda, MD) and was converted into ratios relative to GAPDH. The final relative intensity of XIAP, IL-18, pro-GSDMD, or N-GSDMD was normalized to the control group.

### Reverse transcription PCR (RT-PCR)

Total RNA of RPE-choroid was isolated from pooled rat eye tissues (*N* ≥ 3) using ultRNA Column Purification kit (Applied Biological Materials, Richmond BC, Canada). 200 ng total RNA from each injection group was reverse transcribed into cDNA using the High-Capacity cDNA Reverse Transcription kit (Applied Biosystems, Carlsbad, CA). RT-PCR was carried out on the 7500 Fast Real-time PCR System (Applied Biosystems) using the following cycling conditions: 95 °C for 30 s, 50 °C for 30 s, 72 °C for 30 s, 40 cycles. RT-PCR primer sequences can be found in Table 2. Melting curve analysis was automatically performed right after the cycles’ completion. The results were expressed as mRNA fold-change relative to the control group after normalization to the reference gene, GAPDH, using the 2^−ΔΔCT^ method.

**Table 2:** List of primer sequences

Gene	Forward primer (5′-3′)	Reverse Primer (5′-3′)
X-linked inhibitor of apoptosis (XIAP)	CACACAGTCTACATCTCCT	CTACAACCTGTCCAGTTCT
Glyceraldehyde 3-phosphate dehydrogenase (GAPDH)	CTCTTGTGACAAAGTGGAC	CCATTTGATGTTAGCGGGA

### RPE morphological assessment

To evaluate the morphological changes of RPE cells due to induced inflammasome activity, we developed a custom Photoshop algorithm to measure the area of a set length of RPE monolayer based on RPE pigmentation. For each animal group, a total of nine sections from each animal were used for the analysis. All RPE micrographs were taken under × 60 magnification and subsequently cropped into 12 cm × 2 cm rectangular areas for further processing. Next, choroidal pigments were manually removed and the RPE-only areas were selected by applying “fuzziness”. Then, the pixel count for the cropped area was obtained. The RPE area measurement was expressed as number of pixels. This area measure was equivalent to, but more accurate than measuring the thickness of RPE monolayer.

### RPE cell nuclei count and retinal thickness measurement

Following our established protocol, retinal cross sections within 200 μm distance from the optic disc were chosen for the analysis because of their uniform retinal thickness regardless of embedding orientation [13, 18]. Briefly, RPE nuclei were counted by scanning the whole retinal section under × 20 magnification in 10^3^ μm increments. Retinal thickness was measured from the inner limiting membrane to the photoreceptor outer segments/RPE junction. The mean values of RPE nuclei count per increment, retinal thickness as well as ONL thickness were averaged over a minimum of 4–6 retinal sections per animal at each time point.

### Statistical analyses

Data are presented as mean ± SD. Non-parametric tests were used throughout the study except the vitreal cytokine levels and RT-PCR were analyzed by one-tailed Student’s *t* test. For the non-parametric comparisons between the two groups (Aβ vs control), one-tailed Mann-Whitney *U* tests were used. All analyses were conducted with GraphPad Prism version 6 (GraphPad Software, La Jolla, CA). Statistical significance was set at *p* ≤ 0.05.

## Results

### Pro-inflammatory cytokine secretion in vitreous

**Fig. 1:**
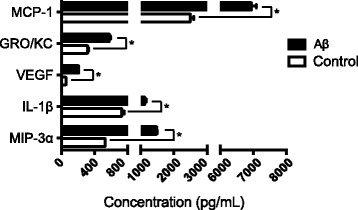
Vitreal cytokine secretion following sequential intraocular injections. An ELISA-based rat cytokine assay was used to measure the vitreal cytokine levels. Cytokines with significant increase in the Aβ-treated eyes compared to the controls were graphed. Sequential injections of Aβ led to a robust increase in chemokines (MCP-1, MIP-3α, GRO/KC), inflammasome product (IL-1β), and growth factor (VEGF). *N* = 7, Student’s *t* test, *p < 0.05

To understand the overall inflammatory status in the rat eyes, we collected and examined the vitreous samples using an ELISA-based cytokine profile assay. We found monocytes chemoattractant protein 1 (MCP-1), chemokine (C-X-C motif) ligand 1 (GRO/KC), vascular endothelial growth factor (VEGF), and macrophage inflammatory protein 3 alpha (MIP-3α) were increased more than 50% in the Aβ injected eyes compared to those in the reverse Aβ injected (hereafter, referred to as “control”) eyes (Fig. 1). A smaller, but significant, increase was associated with vitreal IL-1β, a mature secreted product following NLRP3 inflammasome activation. However, IL-18, another NLRP3 inflammasome product, was downregulated in the vitreous sample from the Aβ injection group compared to the control (Aβ 1142.67 ± 64.15 pg/mL; control 1258.13 ± 56.49 pg/mL), which led us to further look at the inflammasome activity in Aβ-injected rat eyes. Measurements of the additional cytokines studied here are given in Additional file 2: Figure S2.

### NLRP3 inflammasome activation by multiple Aβ injections

**Fig. 2:**
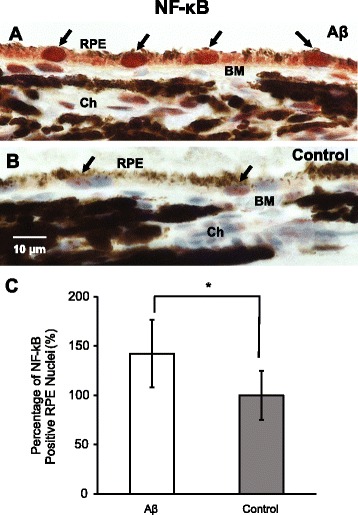
Activation of NF-κB pathway in retinal pigment epithelium (RPE). **a**–**c** In retinal cross sections, injections of Aβ enhanced the nuclear translocalization of NF-κB phosphorylated p65 subunit in RPE (garnet red, arrows, **a**), compared to the light purple RPE nuclei in the control group (arrows, **b**). By counting the number of RPE nuclei with garnet red (AEC) labeling, there was a ~ 50% increase in the positive RPE nuclei over the control group (**c**). BM, Bruch’s membrane; Ch, choroid. Scale bar 10 μm. *N* = 3, Mann-Whitney, *p < 0.05

**Fig. 3:**
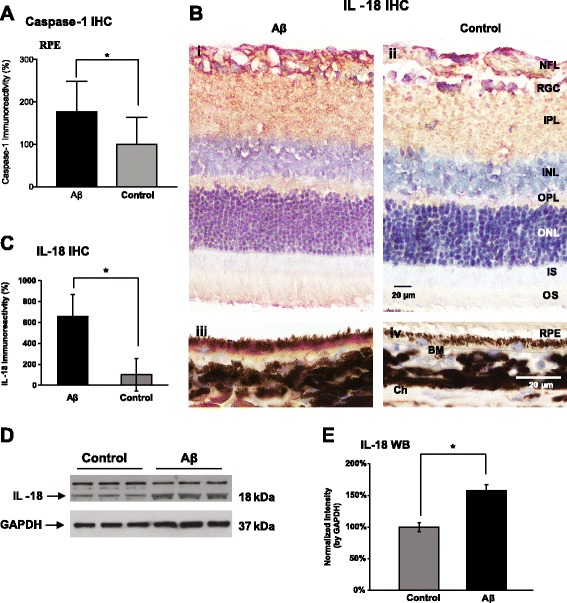
Sequential Aβ injections promote NLRP3 inflammasome activation. **a** Immunoreactivity of total caspase-1 showed a 77% increase in RPE of the Aβ-injected eyes compared to the control group. **b**–**c** The Aβ-injected animals had a 6-fold higher IL-18 immunoreactivity than the control group (**c**). Representative micrographs exhibit an overall more intense IL-18 labeling (AEC, red, panel i and ii), particularly in the RPE layer (iii and iv) (**b**). NFL, nerve fiber layer; RGC, retinal ganglion cells; IPL, inner plexiform layer; INL, inner nuclear layer; OPL, outer plexiform layer; ONL, outer nuclear layer; IS, inner segments; OS, outer segments; RPE, retinal pigment epithelium; BM, Bruch’s membrane; Ch, choroid. Scale bars 20 μm. (**d**–**e**) IL-18 western blotting showed a 58% increase of band intensity (MW = 18 kDa) in Aβ injected animals compared to the controls. Bands shown are technical triplicates of the same-pooled sample. *N* ≥ 3, Mann-Whitney, *p < 0.05

NLRP3 inflammasome activation is achieved by a sequential stimulation of a priming signal and an activation signal. To examine inflammasome activation in RPE, we first looked at the nuclear translocalization of NF-κB, a signature event of NF-κB activation. Using an antibody specifically targeting the phosphorylated p65 subunit of NF-κB, we found strong immunoreactivity in the nuclei of RPE cells in Aβ-injected eyes compared to control eyes (Fig. 2a–c). Next, we immunolabeled caspase-1 in the RPE layer of both Aβ-injected and control eyes, revealing that caspase-1 immunoreactivity was enhanced by 77% compared to the control eyes (Fig. 3a). Consistent with the elevation of IL-1β in the vitreous, we found the other inflammasome activation product, IL-18, was also upregulated, showing more than 6-fold higher immunoreactivity in the RPE layer of Aβ-injected eyes (Fig. 3b–c) and a 58% increase of IL-18 band intensity in protein lysates from the Aβ injected eyes, compared to the control eyes (Fig. 3d–e).

### Cell death pathways triggered by Aβ injections

**Fig. 4:**
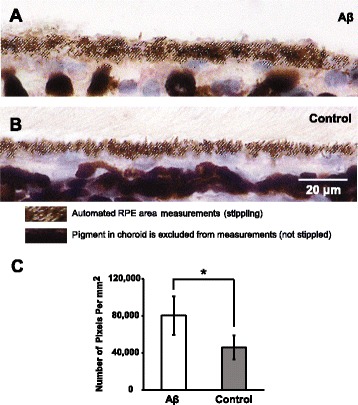
RPE morphological changes following sequential Aβ injections. **a** Sequential Aβ injections caused RPE swelling, which was quantified by RPE area measurements using a custom Photoshop algorithm identifying the stippled area of RPE pigment. **b** Animals receiving sequential control solution injections possessed thinner RPE layer compared to the Aβ group, indicated by the stippled area of RPE pigment. **c** The number of pixels is a surrogate marker for RPE area measurement, which showed about 2-fold increase in Aβ group compared to the control group. *N* = 3, Mann-Whitney, *p < 0.05

To study the forms of RPE cell death often seen in late AMD, especially GA, we next analyzed morphological changes in RPE after Aβ injections. Using a custom Photoshop algorithm, we first applied a pigment threshold to identify the area occupied by RPE within a standard 12 cm × 2 cm boxed area centered on the RPE monolayer. Next, the number of pixels within the pigment threshold of that area was obtained as an index of the area measurement occupied by the RPE. Higher pixel values indicated thicker RPE monolayers. By comparing the Aβ-injected to the control groups, we observed a 2-fold increase in the number of pixels, suggesting a significant area increase in the RPE, presumably due to swelling of the RPE cells (Fig. 4).

**Fig. 5:**
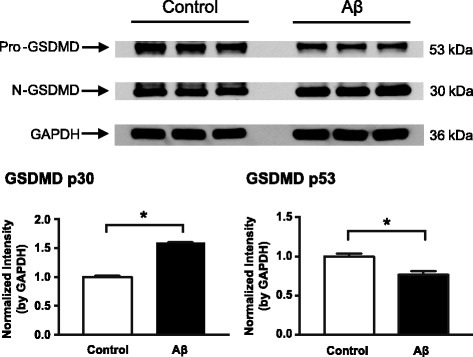
Activation of GSDMD mediated pyroptosis in response to Aβ stimulation. RPE-choroid protein lysates from animals receiving either Aβ or control injections were probed with a GSDMD antibody. Compared to the control samples, Aβ significantly promoted the proteolytic cleavage of pro-GSDMD (53 kDa) into N-terminal GSDMD fragment (N-GSDMD, 30 kDa), a signature event indicating the activation of the pyroptotic pathway. GAPDH served as a loading control (36 kDa). Bands shown are technical triplicates of the same-pooled sample. *N* ≥ 3, Mann-Whitney, *p < 0.05

To understand what caused the RPE to enlarge or swell, we prepared RPE-choroid tissue homogenates from both injection groups and tested them for gasdermin D (GSDMD), a protein executing pyroptosis and whose proteolytic cleavage is triggered by both canonical and non-canonical inflammasome activation in immune cells [19–21]. The cleaved GSDMD N-terminal fragment (N-GSDMD) at 30 kDa was increased, while the uncleaved pro-GSDMD full-length protein at 53 kDa was decreased, in the Aβ group compared to the control group (Fig. 5).

**Fig. 6:**
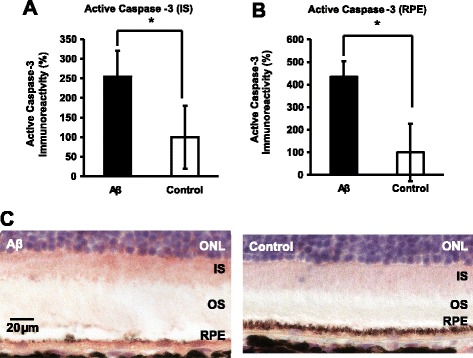
Caspase-3 activation in the outer retina of Aβ-stimulated eyes. **a**–**b** Immunoreactivity of active/cleaved caspase-3 was 2.5- and 4.5-fold higher in the Aβ-injected eyes for photoreceptors’ inner segments (IS) and RPE, respectively, compared to the control eyes. *N* = 3, Mann-Whitney, *p < 0.05. **c** Representative micrographs illustrated examples of the semi-quantitative grading of active caspase-3’s immunoreactivity in both Aβ-injected and control animals. Robust caspase-3 immunoreactivity in the IS was seen in the Aβ-injected retina, whereas weak and intermediate caspase-3 immunoreactivity was seen in the control eyes. ONL, outer nuclear layer; OS, outer segments. Scale bar 20 μm

**Fig. 7:**
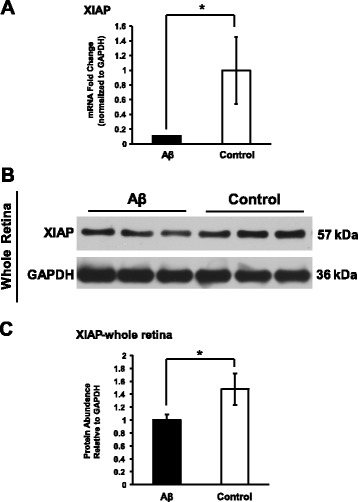
Decline of XIAP gene expression levels in Aβ-stimulated eyes. **a** Reverse transcriptional PCR revealed a 90% reduction of XIAP mRNA in Aβ-stimulated eyes compared to controls. *N* ≥ 3, Student’s *t* test, *p < 0.05. **b**–**c** In whole retina protein lysates, there was a significant 33% decrease of the XIAP protein level in Aβ-stimulated eyes compared to reverse Aβ controls. Bands shown are technical triplicates of the same-pooled sample. *N* = 5, Mann-Whitney, *p < 0.05

Next, we also tested for apoptosis involvement using caspase-3 activation as a surrogate marker. By immunohistochemistry, we found 2.5- to 4.5-fold higher immunoreactivity levels of active caspase-3 in photoreceptor inner segments and RPE of the Aβ group, respectively, compared to the control group (Fig. 6a–b). Two examples of active caspase-3 immunoreactivity from both Aβ-injected and control eyes were provided to demonstrate the enhanced red AEC labeling in photoreceptor inner segments and RPE from Aβ-injected eyes (Fig. 6c). To further support this finding, we measured the mRNA and protein levels of X-chromosome-linked inhibitor of apoptosis (XIAP), a classic anti-apoptosis factor. At the transcriptional level, there was a 10-fold reduction in the Aβ group compared to the controls (Fig. 7a). Concomitant with the mRNA data, there was a 33% reduction in XIAP protein compared to the control group (Fig. 7b–c).

**Fig. 8:**
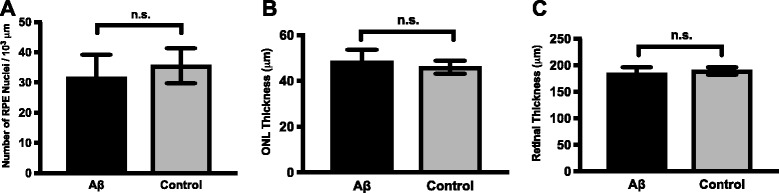
No changes in RPE nuclei number and retinal thickness in Aβ-stimulated eyes. The number of RPE nuclei per 10^3^ μm (**a**), ONL thickness (**b**), and retinal thickness (**c**) were measured in both the Aβ-stimulated and control animals’ eyes. No significant difference was found between the two groups in all three analytical categories (*N* = 3, Mann-Whitney)

Finally, to determine whether the activation of pyroptotic and apoptotic pathways caused overt anatomical changes to the retina, we counted the RPE nuclei and assessed retinal thickness. Our results showed that there was no significant RPE nuclei loss or retinal thickness changes, including the ONL thickness, in the retinal cross sections of Aβ-injected eyes compared to the control eyes (Fig. 8). These measurements indicated no overt anatomical/histological changes were evident at the time point studied here.

## Discussion

In late stage AMD, RPE cells die, yet the cell death pathways responsible remain a mystery. Based on pathological specimens of AMD donor eyes, atrophic RPE cells were thought to die from a necrotic cell death pathway, in which hypopigmented RPE cells filled with membrane-bound melanolipofuscin were eliminated, resulting in the increased pigmentation and cell body enlargement in adjacent RPE cells [4]. More recently, reports from Kaneko et al. [5] and Tarallo et al. [6] highlight other cell death mechanisms (such as apoptosis and pyroptosis) that also likely contribute to atrophic RPE demise. However, the question remains as to what factors initiate the cell death cascades in AMD. In this study, using an animal model in which we mimicked pro-inflammation due to prolonged (14 days) exposure to a drusen component, Aβ, we assessed the level of involvement of pyroptotic and apoptotic pathways.

### RPE inflammasome activation is a feature of the model

NLRP3 inflammasome activation, one of the fundamental innate immune defense mechanisms, has recently been studied for its role in the development of AMD [22]. Our earlier study indicated that a single Aβ injection resulted in a peak in pro-inflammation at 4 days post injection, but then dramatically subsided [13]. In the current study, we extended the duration of pro-inflammation from 4 to 14 days by making sequential injections of Aβ every 4 days to achieve better retinal penetration and mimic a chronic inflammatory microenvironment in the outer retina (Additional file 3: Figure S3). Our results demonstrated that longer exposure to pro-inflammation triggered robust NF-κB p65 subunit translocalization in RPE nuclei, elevated levels of total caspase-1 immunoreactivity, and enhanced secretion of IL-1β in vitreous and increased IL-18 presence in retina. Collectively, these results support inflammasome activity in the RPE (Fig. 3, Additional file 3: Figure S3). When compared with other inflammasome studies on RPE cells including ours, the current study demonstrated significant increase of IL-18 in neuroretina and RPE-choroid, but not in the vitreous where IL-1β was found upregulated by more than 50% (Figs. 1 and 3, Additional file 2: Figure S2). This secretion pattern is unique in that it diversifies the downstream biological events of these inflammasome cytokines: in our previous acute model of single Aβ injection, vitreal IL-18 increased by more than 3-fold at day 14 whereas vitreal IL-1β elevated by less than 50% compared to the controls [13]. Such discrepancy in IL-1β/18 vitreal levels can be partially explained by the “distinct licensing requirements” for processing these two cytokines by NLRP3 inflammasomes, which might involve further regulation of caspase-11 and/or reactive oxygen species (ROS), as evident in immune cells [23]. This mechanism is also very likely to be true in RPE cells since Aβ has recently been shown to induce NLRP3 inflammasome activation via NADPH oxidase- and mitochondria-dependent ROS pathway in vitro [24]. Other studies suggest that a high level of retinal IL-18 (including RPE-choroid) is more related to the cell death events in GA [6, 25], which support our findings that higher IL-18 immunoreactivity levels in Aβ-injected retina are correlated with the activation of pyroptotic pathway.

Sequential injections of Aβ also provide a good model to study Aβ’s role in inflammasome activation in the eye. Considered as one of the pathological hallmarks in AD, the deposition of Aβ in the AD brain is associated with elevated NLRP3 inflammasome activity, particularly the elevation of IL-1β production [26–28]. Using microglia culture models, Halle et al. demonstrated the importance of NLRP3 inflammasome activation for the recruitment of microglia to Aβ deposits in the AD brain [29]. Heneka et al. further discovered that in the absence of NLRP3 or caspase-1, mice carrying mutations associated with familial AD were largely protected from spatial memory loss, and demonstrated reduced IL-1β secretion and enhanced Aβ clearance [27]. However, the involvement of these pathways has not been well established in the eye tissue. As a continuation from our previous studies [13], our current Aβ multi-injection model recapitulates seminal features associated with each step of the NLRP3 inflammasome activation cascades. Therefore, by giving the animals sequential Aβ injections, we were able to sustain Aβ-induced pro-inflammatory responses in the neuroretina and RPE at a comparably high level until day 14, when the presence of Aβ was much diminished in the single Aβ injection model [13].

### Pyroptosis and apoptosis may contribute to RPE cell death in this model

The involvement of NLRP3 inflammasome activation in RPE has been studied in many different AMD models [30–33]. However, once the inflammasome is activated, little is known of the exact biological events that occur and whether these events lead to cell death. Using rodent and non-human primate models, Doyle and colleagues demonstrate the efficacy of IL-18 treatment as a potential alternative, adjuvant therapy for CNV [34–36]. On the other side, Ambati and collaborators showed evidence that IL-18 drives RPE degeneration after NLRP3 inflammasome activation in murine models [6, 37]. In the current study, we also assessed the changes after activation of the NLRP3 inflammasome by Aβ. Intriguingly, after prolonged pro-inflammation in outer retina, we found enlarged or swollen RPE cells and significant increases in the proteolytic cleavage of full-length GSDMD in the RPE-choroid tissues. As reported by earlier studies, the cytolytic effects of pyroptosis are mediated by the oligomerization of the GSDMD’s N-terminal fragments (N-GSDMD) in cellular membrane, resulting in the cell-burst pore formation [38]. Hence, it confirms the activation of pyroptotic pathway in the Aβ-injected animals [19]. Therefore, we have demonstrated the co-existence of two events that occur after inflammasome activation: the secretion of mature pro-inflammatory cytokines, including the inflammasome products (IL-18 and IL-1β) and morphological and western blot evidence that supports the GSDMD-mediated pyroptotic pathway activation in RPE cells. Such an orchestrated response has also been seen in non-ocular cell types [39].

Unlike pyroptosis, which rapidly lyses the cell, apoptosis is considered as a non-inflammatory and non-cytolytic form of programmed cell death. From a canonical point of view, pyroptosis is biochemically characterized as caspase-1 dependent and caspase-3 independent, whereas apoptosis is often caspase-3 dependent and caspase-1 independent. Interestingly, in the current study, we observed parallel cleavage of both caspase-1 and caspase-3 in the RPE tissue, challenging the dogma of their mutual exclusiveness. Although it is biologically impossible for one single cell to undergo both distinctive cell death pathways, it is still likely for one type of tissue, such as the RPE monolayer in the retina, to accommodate these pathways in a spatially discrete and stimulus dose-dependent manner. One study using murine bone marrow-derived macrophages exploited the potential of crosstalk between pyroptosis and apoptosis. The authors exhibited a DNA-dose dependent integral model for cell death, with apoptosis seen at a lower-dose of DNA stimulation and pyroptosis at higher doses. They further concluded that such an explicit response is regulated through caspase-8 activation [40].

Despite the fact that our data supported the activation of both pyroptotic and apoptotic pathways in the RPE-choroid tissue of multiple Aβ-injected eyes, there were no significant anatomical changes as indicated by RPE nuclei counts and retinal thickness measurements (Fig. 8). All the biochemical markers used in this study (IL-18, IL-1β, NF-κB, caspase-1, caspase-3, GSDMD, XIAP) proved changes leading towards RPE atrophy via both cell death pathways. Combined with the anatomical data, our results suggest a transition between early and middle-to-late stage of dry AMD, where RPE cells begin to show early biochemical signs of cell death and before any observable retinal structural changes.

### RPE-specific vs choroidal macrophage-mediated responses in vivo

The ability to dissect RPE-choroid from neuroretina was demonstrated in our earlier work [11, 13] and those of others [41]. However, it is possible that in the RPE-choroid sample, the choroidal component with macrophages, may also contribute to the inflammasome activity in our in vivo work. Testing for activated macrophages by immunohistochemistry in paraffin sections or by microdissection of choroidal tissue alone [42] may help us better understand the migration of immune cells from choroid in our Aβ-injected animal groups. It is possible that the choroidal macrophages, important immune cells associated with AMD [43, 44] in combination with RPE may both work to exacerbate the chronic inflammatory milieu in the AMD eye. Therefore, future in vitro work on cultured RPE cells and choroidal macrophages will allow us to define the role of each cell type without the confounding effects from the other.

## Conclusions

In conclusion, we have demonstrated the activation of two distinct cell death pathways in RPE following prolonged pro-inflammation induced by drusen component, Aβ. For the first time, we show that GSDMD cleavage is associated with inflammasome activation in RPE, providing the molecular basis for pyroptosis participation in this model. Consistent with other studies using postmortem human AMD donor eye tissues [6], our model recapitulates the key events in the NLRP3 inflammasome cascade. Furthermore, the presence of swollen, enlarged RPE cells in this model represents another prominent feature of RPE morphological changes during AMD progression [10, 45]. Even though the retinal morphological analysis found no evidence of ultimate RPE cell loss or retinal thinning in this model, the biochemical events revealed here point towards a critical transitional stage that may further lead to the occurrence of RPE cell death. Understanding the detailed molecular mechanisms associated with this stage will benefit future endeavors in the search of therapeutic agents to slow down, or even prevent, RPE cell death in AMD.

## Additional file

Additional file 1: Figure S1. Preparation of oligomeric Aβ. (A) Various incubation conditions were tested for the optimal oligomeric Aβ generation. Small oligomeric (dimeric and trimeric) and monomeric Aβ species were generated after a shorter period of incubation time (24 h, 100 μM). Note that the higher molecular Aβ species began to increase after 48 h (MW > 170 kDa). (B) Western blot of the 48 h-incubated oligomeric Aβ solution, prepared for intraocular injections (7 μg/5 μL). The control peptide was prepared the same way as Aβ, but was non-reactive to the 6E10 antibody detection. (EPS 5439 kb)
Additional file 2: Figure S2. Additional vitreal cytokine levels following sequential Aβ injections. A list of rat cytokines in addition to Fig. 1 was examined in vitreous samples from both the Aβ-stimulated and the control animals’ eyes using the ELISA-based premade assay. Unlike Fig. 1’s data, all these cytokines showed less than 50% change of concentration levels between the two animal groups, many of which had even lower measurements in the Aβ-stimulated group, for instance, IL-18. *N* = 7, Student’s *t* test, **p < 0.05*. RANTES, regulated on activation normal T cell expressed and secreted; GM-CSF, granulocyte macrophage colony stimulating factor; EPO, erythropoietin; G-CSF, granulocyte colony stimulating factor; M-CSF, macrophage colony stimulating factor. (EPS 170 kb)
Additional file 3: Figure S3. Retinal distribution of injected Aβ. To ensure the effectiveness of sequential Aβ injections, retinal cross-sections from day 14 (indicated as “3X Aβ day 14”) were stained for the injected Aβ using the 6E10 antibody (Table 1). As visualized by the red AEC, Aβ exhibited good penetration of all retinal layers from retinal ganglion cell layer to RPE. Compared to its counterpart from our previous acute Aβ model (indicated as “1X Aβ day 14”), the current 3X Aβ day 14 retinal cross-sections showed much stronger Aβ immunoreactivity, suggesting a higher retinal Aβ concentration maintained by the sequential Aβ injections. Such robust Aβ immunoreactivity in the 3X Aβ day 14 tissue (neuroretina + RPE) was comparable to those of 1X Aβ retinal tissues at days 1 and 4, with even more enhanced labeling in the outer nuclei layer. RGC, retinal ganglion cell; IPL, inner plexiform layer; INL, inner nuclei layer; OPL, outer plexiform layer; ONL, outer nuclei layer; IS/OS, inner/outer segments. Scale bar 20 μm. (EPS 13727 kb)

## Abbreviations

AD: Alzheimer’s disease
AEC: 3-amino-9-ethylcarbazole
AMD: Age-related macular degeneration
ASC: Apoptosis-associated speck-like protein containing a carboxy-terminal CARD
Aβ: Amyloid beta
CNV: Choroidal neovascularization
DMSO: Dimethyl sulfoxide
ELISA: Enzyme-linked immunosorbent assay
GA: Geographic atrophy
GRO/KC: Chemokine (C-X-C motif) ligand 1
GSDMD: Gasdermin D
HFIP: 1,1,1,3,3,3-hexafluoro-2-propanol
IL-18: Interleukin-18
IL-1β: Interleukin-1beta
MCP-1: Monocytes chemoattractant protein 1
MIP-3α: Macrophage inflammatory protein 3 alpha
NF-κB: Nuclear factor kappa B
N-GSDMD: GSDMD N-terminal fragment
NLRP3: NLR family pyrin domain containing 3
PBS: Phosphate buffered saline
PCR: Polymerase chain reaction
RIPA: Radioimmunoprecipitation assay
RPE: Retinal pigment epithelium
TUNEL: Terminal deoxynucleotidyl transferase dUTP nick end-labeling
VEGF: Vascular endothelial growth factor
WB: Western blot
XIAP: X-chromosome linked inhibitor of apoptosis

## References

[1] Wong WL, Su X, Li X, Cheung CM, Klein R, Cheng CY, Wong TY (2014). Global prevalence of age-related macular degeneration and disease burden projection for 2020 and 2040: a systematic review and meta-analysis. Lancet Glob Health.

[2] Age-Related Eye Disease Study Research G (2005). A simplified severity scale for age-related macular degeneration: AREDS report no. 18. Arch Ophthalmol.

[3] Amoaku WM, Chakravarthy U, Gale R, Gavin M, Ghanchi F, Gibson J, Harding S, Johnston RL, Kelly SP, Lotery A (2015). Defining response to anti-VEGF therapies in neovascular AMD. Eye (Lond).

[4] Sarks JP, Sarks SH, Killingsworth MC (1988). Evolution of geographic atrophy of the retinal pigment epithelium. Eye (Lond).

[5] Kaneko H, Dridi S, Tarallo V, Gelfand BD, Fowler BJ, Cho WG, Kleinman ME, Ponicsan SL, Hauswirth WW, Chiodo VA (2011). DICER1 deficit induces Alu RNA toxicity in age-related macular degeneration. Nature.

[6] Tarallo V, Hirano Y, Gelfand BD, Dridi S, Kerur N, Kim Y, Cho WG, Kaneko H, Fowler BJ, Bogdanovich S (2012). DICER1 loss and Alu RNA induce age-related macular degeneration via the NLRP3 inflammasome and MyD88. Cell.

[7] Hageman GS, Luthert PJ, Victor Chong NH, Johnson LV, Anderson DH, Mullins RF (2001). An integrated hypothesis that considers drusen as biomarkers of immune-mediated processes at the RPE-Bruch’s membrane interface in aging and age-related macular degeneration. Prog Retin Eye Res.

[8] Dunaief JL, Dentchev T, Ying GS, Milam AH (2002). The role of apoptosis in age-related macular degeneration. Arch Ophthalmol.

[9] Bergsbaken T, Fink SL, Cookson BT (2009). Pyroptosis: host cell death and inflammation. Nat Rev Microbiol.

[10] Gao J, Liu RT, Cao S, Cui JZ, Wang A, To E, Matsubara JA (2015). NLRP3 inflammasome: activation and regulation in age-related macular degeneration. Mediat Inflamm.

[11] Zhao T, Gao J, Van J, To E, Wang A, Cao S, Cui JZ, Guo JP, Lee M, McGeer PL, Matsubara JA (2015). Age-related increases in amyloid beta and membrane attack complex: evidence of inflammasome activation in the rodent eye. J Neuroinflammation.

[12] Hoh Kam J, Lenassi E, Jeffery G. Viewing ageing eyes: diverse sites of amyloid Beta accumulation in the ageing mouse retina and the up-regulation of macrophages. PLoS One. 2010;5(10):e13127. 10.1371/journal.pone.0013127 PMC2948519 20957206

[13] Liu RT, Gao J, Cao S, Sandhu N, Cui JZ, Chou CL, Fang E, Matsubara JA (2013). Inflammatory mediators induced by amyloid-beta in the retina and RPE in vivo: implications for inflammasome activation in age-related macular degeneration. Invest Ophthalmol Vis Sci.

[14] Kurji KH, Cui JZ, Lin T, Harriman D, Prasad SS, Kojic L, Matsubara JA (2010). Microarray analysis identifies changes in inflammatory gene expression in response to amyloid-beta stimulation of cultured human retinal pigment epithelial cells. Invest Ophthalmol Vis Sci.

[15] Liu RT, Wang A, To E, Gao J, Cao S, Cui JZ, Matsubara JA (2014). Vinpocetine inhibits amyloid-beta induced activation of NF-kappaB, NLRP3 inflammasome and cytokine production in retinal pigment epithelial cells. Exp Eye Res.

[16] Sarroukh R, Cerf E, Derclaye S, Dufrene YF, Goormaghtigh E, Ruysschaert JM, Raussens V (2011). Transformation of amyloid beta(1-40) oligomers into fibrils is characterized by a major change in secondary structure. Cell Mol Life Sci.

[17] Stine WB, Jungbauer L, Yu C, LaDu MJ (2011). Preparing synthetic Abeta in different aggregation states. Methods Mol Biol.

[18] Guo L, Normando EM, Nizari S, Lara D, Cordeiro MF (2010). Tracking longitudinal retinal changes in experimental ocular hypertension using the cSLO and spectral domain-OCT. Invest Ophthalmol Vis Sci.

[19] Shi J, Zhao Y, Wang K, Shi X, Wang Y, Huang H, Zhuang Y, Cai T, Wang F, Shao F (2015). Cleavage of GSDMD by inflammatory caspases determines pyroptotic cell death. Nature.

[20] He WT, Wan H, Hu L, Chen P, Wang X, Huang Z, Yang ZH, Zhong CQ, Han J (2015). Gasdermin D is an executor of pyroptosis and required for interleukin-1beta secretion. Cell Res.

[21] Kayagaki N, Stowe IB, Lee BL, O'Rourke K, Anderson K, Warming S, Cuellar T, Haley B, Roose-Girma M, Phung QT (2015). Caspase-11 cleaves gasdermin D for non-canonical inflammasome signalling. Nature.

[22] Celkova L, Doyle SL, Campbell M (2015). NLRP3 inflammasome and pathobiology in AMD. J Clin Med.

[23] Schmidt RL, Lenz LL (2012). Distinct licensing of IL-18 and IL-1beta secretion in response to NLRP3 inflammasome activation. PLoS One.

[24] Wang K, Yao Y, Zhu X, Zhang K, Zhou F, Zhu L. Amyloid β induces NLRP3 inflammasome activation in retinal pigment epithelial cells via NADPH oxidase- and mitochondria-dependent ROS production. J Biochem Mol Toxicol. 2016;31:e21887. 10.1002/jbt.21887 28004443

[25] Kim Y, Tarallo V, Kerur N, Yasuma T, Gelfand BD, Bastos-Carvalho A, Hirano Y, Yasuma R, Mizutani T, Fowler BJ (2014). DICER1/Alu RNA dysmetabolism induces caspase-8-mediated cell death in age-related macular degeneration. Proc Natl Acad Sci U S A.

[26] Olsen I, Singhrao SK (2016). Inflammasome involvement in Alzheimer’s disease. J Alzheimers Dis.

[27] Heneka MT, Kummer MP, Stutz A, Delekate A, Schwartz S, Vieira-Saecker A, Griep A, Axt D, Remus A, Tzeng TC (2013). NLRP3 is activated in Alzheimer’s disease and contributes to pathology in APP/PS1 mice. Nature.

[28] Salminen A, Ojala J, Suuronen T, Kaarniranta K, Kauppinen A (2008). Amyloid-beta oligomers set fire to inflammasomes and induce Alzheimer’s pathology. J Cell Mol Med.

[29] Halle A, Hornung V, Petzold GC, Stewart CR, Monks BG, Reinheckel T, Fitzgerald KA, Latz E, Moore KJ, Golenbock DT (2008). The NALP3 inflammasome is involved in the innate immune response to amyloid-beta. Nat Immunol.

[30] Tseng WA, Thein T, Kinnunen K, Lashkari K, Gregory MS, D’Amore PA, Ksander BR (2013). NLRP3 inflammasome activation in retinal pigment epithelial cells by lysosomal destabilization: implications for age-related macular degeneration. Invest Ophthalmol Vis Sci.

[31] Anderson OA, Finkelstein A, Shima DT (2013). A2E induces IL-1ss production in retinal pigment epithelial cells via the NLRP3 inflammasome. PLoS One.

[32] Kerur N, Hirano Y, Tarallo V, Fowler BJ, Bastos-Carvalho A, Yasuma T, Yasuma R, Kim Y, Hinton DR, Kirschning CJ (2013). TLR-independent and P2X7-dependent signaling mediate Alu RNA-induced NLRP3 inflammasome activation in geographic atrophy. Invest Ophthalmol Vis Sci.

[33] Kauppinen A, Niskanen H, Suuronen T, Kinnunen K, Salminen A, Kaarniranta K (2012). Oxidative stress activates NLRP3 inflammasomes in ARPE-19 cells—implications for age-related macular degeneration (AMD). Immunol Lett.

[34] Doyle SL, Campbell M, Ozaki E, Salomon RG, Mori A, Kenna PF, Farrar GJ, Kiang AS, Humphries MM, Lavelle EC (2012). NLRP3 has a protective role in age-related macular degeneration through the induction of IL-18 by drusen components. Nat Med.

[35] Doyle SL, Ozaki E, Brennan K, Humphries MM, Mulfaul K, Keaney J, Kenna PF, Maminishkis A, Kiang AS, Saunders SP (2014). IL-18 attenuates experimental choroidal neovascularization as a potential therapy for wet age-related macular degeneration. Sci Transl Med.

[36] Doyle SL, Lopez FJ, Celkova L, Brennan K, Mulfaul K, Ozaki E, Kenna PF, Kurali E, Hudson N, Doggett T (2015). IL-18 immunotherapy for neovascular AMD: tolerability and efficacy in nonhuman primates. Invest Ophthalmol Vis Sci.

[37] Hirano Y, Yasuma T, Mizutani T, Fowler BJ, Tarallo V, Yasuma R, Kim Y, Bastos-Carvalho A, Kerur N, Gelfand BD (2014). IL-18 is not therapeutic for neovascular age-related macular degeneration. Nat Med.

[38] Liu X, Zhang Z, Ruan J, Pan Y, Magupalli VG, Wu H, Lieberman J (2016). Inflammasome-activated gasdermin D causes pyroptosis by forming membrane pores. Nature.

[39] Sborgi L, Ruhl S, Mulvihill E, Pipercevic J, Heilig R, Stahlberg H, Farady CJ, Muller DJ, Broz P, Hiller S (2016). GSDMD membrane pore formation constitutes the mechanism of pyroptotic cell death. EMBO J.

[40] Sagulenko V, Thygesen SJ, Sester DP, Idris A, Cridland JA, Vajjhala PR, Roberts TL, Schroder K, Vince JE, Hill JM (2013). AIM2 and NLRP3 inflammasomes activate both apoptotic and pyroptotic death pathways via ASC. Cell Death Differ.

[41] Woodell A, Coughlin B, Kunchithapautham K, Casey S, Williamson T, Ferrell WD, Atkinson C, Jones BW, Rohrer B (2013). Alternative complement pathway deficiency ameliorates chronic smoke-induced functional and morphological ocular injury. PLoS One.

[42] Newman AM, Gallo NB, Hancox LS, Miller NJ, Radeke CM, Maloney MA, Cooper JB, Hageman GS, Anderson DH, Johnson LV, Radeke MJ (2012). Systems-level analysis of age-related macular degeneration reveals global biomarkers and phenotype-specific functional networks. Genome Med.

[43] Ardeljan D, Chan CC (2013). Aging is not a disease: distinguishing age-related macular degeneration from aging. Prog Retin Eye Res.

[44] McLeod DS, Bhutto I, Edwards MM, Silver RE, Seddon JM, Lutty GA (2016). Distribution and quantification of choroidal macrophages in human eyes with age-related macular degeneration. Invest Ophthalmol Vis Sci.

[45] Bressler SB, Bressler, N.M.: Age-related macular degeneration: non-neovascular early AMD, intermediate AMD, and geographic atrophy**.** In Retina (Ryan SJ ed., vol. 2, 5th edition. London: Elsevier; 2014. p. 1150–82.

